# Non-linear relationships between density and demographic traits in three *Aedes* species

**DOI:** 10.1038/s41598-022-11909-y

**Published:** 2022-05-16

**Authors:** Logan A. Sauers, Kelsey E. Hawes, Steven A. Juliano

**Affiliations:** School of Biological Sciences, Illinois State University, 251 S. School St., Normal, IL 61761 USA

**Keywords:** Ecology, Community ecology, Population dynamics

## Abstract

Understanding the relationship of population dynamics to density is central to many ecological investigations. Despite the importance of density-dependence in determining population growth, the empirical relationship between density and *per capita* growth remains understudied in most systems and is often assumed to be linear. In experimental studies of interspecific competition, investigators often evaluate the predicted outcomes by assuming such linear relationships, fitting linear functions, and estimating parameters of competition models. In this paper, we experimentally describe the shape of the relationship between estimated population rate of change and initial density using laboratory-reared populations of three mosquito species. We estimated *per capita* growth rate for these experimental populations over a 30-fold range of larval densities at a standard resource abundance. We then compared fits of linear models and several different nonlinear models for the relationship of estimated rate of change and density. We find that that the relationship between density and *per capita* growth is strongly non-linear in *Aedes aegypti* (Linnaeus), *Aedes albopictus* (Skuse), *and Aedes triseriatus* (Say) mosquitoes. Components of population growth (survivorship, development time, adult size) are also nonlinearly related to initial density. The causes and consequences of this nonlinearity are likely to be important issues for population and community ecology.

## Introduction

A population’s rate of change is central to theory and empirical studies of ecology^1–5^, evolution^6–8^, and disease transmission^9,10^. Often population growth is affected by population density^11^; thus, density dependent effects on population rate of change are central to understanding population dynamics. Density dependence may result from resource competition^12,13^, availability of mates^14^, social interactions^15^, or dispersal^16^. Resource limitation is likely the most postulated process producing density dependent effects via increasing death rates, decreasing birth rates, or decreasing individual growth rates^17,18^. Ultimately, when these density dependent effects act on individuals the population’s *per capita* growth rate, the average rate at which individuals contribute to change of population size (dN/Ndt), is affected^1^. Such density dependent effects, acting on individuals, ultimately influence *per capita* growth rate^19,20^.

Understanding density dependent effects on population growth can be especially important for investigations of disease vectors^18,21–26^. Vectors are often targets of control efforts that may change population density, and thus may alter survival, growth, development, or fecundity^27,28^. For vector mosquitoes, both field and laboratory studies indicate that density dependent effects on larvae can be strong, and can impact survival, individual growth rate (and resulting size and fecundity), and development rate (and resulting age at first reproduction) (e.g.^21,23,24,29–35^).

Ecological models of population *per capita* rate of change, such as Verhulst’s logistic or Lotka-Volterra models, postulate a linear relationship of *per capita* growth to intra- or inter-specific density^1,36,37^; however, for most organisms the relationship between density and *per capita* growth rate is not well known^38–40^, and a linear relationship is only one possibility. Many of the relationships between density and the demographic components of population dynamics (e.g., survivorship, fecundity, reproductive age) are non-linear^36^. Multiple studies point out the apparent flaw in the assumption of linearity, and among the few studies explicitly testing the form of the relationship between density and *per capita* growth rates, nonlinearity has been documented repeatedly (e.g.,^11,37,41–43^). Additionally, individual based models of resource competition suggest that multiple aspects of species biology can produce nonlinear relationships of *per capita* growth to population density^4^. Whether relationships between *per capita* growth and population density are linear is an important question, as nonlinearity would indicate that the effects of population density and resource use on population growth rate are not uniform across density (as they are modeled in simple logistic growth). Thus, the effects of increasing density may depress growth most strongly at low or high densities, and therefore may relate to rates of population recovery from low density^11,44^.

Determining the relationship between population density and *per capita* growth is likely to improve predictions and models of vector populations. Models assuming linear effects of intraspecific density on *per capita* rate of increase have been used to estimate logistic growth models of vector mosquitoes^36,45^, or to test theoretical predictions about responses to mortality^5,47^. Models assuming linear effects of both intra- and interspecific densities have been used often to evaluate potential for coexistence of competing vector species^24,47–53^. Among these investigations, only^51^ tested explicitly alternative models for the relationship of estimated growth rate to densities; they found the best model to be one with linear relationships of estimated finite *per capita* rate of increase to density for *A. aegypti.*

To investigate the relationship between density and *per capita* growth we use three important vector species: *Aedes aegypti* (Linnaeus)*, Aedes albopictus* (Skuse), and *Aedes triseriatus* (Say). These container-dwelling mosquitoes are an excellent model system for investigating density dependent population dynamics because they are often impacted by density dependent effects in nature (e.g.,^21,29,33–35,54^). As container-dwelling mosquitoes they are easily reared in the laboratory in conditions that realistically simulate natural environments. All three have often been the subject of population studies using Livdahl and Sugihara’s composite index of performance^36^, which uses a life table approach to estimate *per capita* rate of change for large experimental cohorts^36^. Use of Livdahl and Sugihara’s index facilitates rearing of high-density cohorts with substantial replication because it removes the necessity of following adult females for their entire lives to obtain *per capita* rate of increase from a full life table^36^. Two separate laboratory studies have shown that this index is highly correlated with *per capita* rate of increase estimated from a full life table following reproduction of 100% of a cohort of females^43,55^. Finally, all three species are important vectors of human diseases and targets of control efforts that may alter densities of larvae, thus altering density-dependent effects on population growth, production of adults, and other traits relevant to vectorial capacities^26^.

Here, we investigate the relationship between larval density and the estimated *per capita* rate of increase as calculated by the index of performance, and the component variables that are used to calculate the index: survivorship to adulthood; adult female development time; and adult female size as a predictor of female fecundity. The primary objective was to determine whether the relationship of estimated *per capita* rate of increase to larval density is best fit by a linear logistic growth model, or alternative nonlinear models: θ logisitic; Gompertz; or polynomial. We hypothesized that the relationship between density and *per capita* growth is in fact non-linear. Additionally, we tested for nonlinearities in the relationships of component variables (survivorship, development time, adult size) to larval density and evaluated how those components may contribute to any nonlinear relationship observed for estimated *per capita* rate of increase.

## Methods

### Laboratory populations and rearing

All colonies originated from field collected larvae (origins in Table S1) and had been maintained in the laboratory for several generations. Colony larvae were raised in 30 × 15 cm pans and fed bovine liver powder weekly. Colonies of *A. triseriatus* and *A. aegypti* were raised in 0.6 m^3^ screened cages. These containers were held in an insectary at ~ 24 °C with a 17:7 h L:D cycle and an 0.75 h dawn or dusk phase at the beginning and end of the light phase. *Aedes albopictus* were raised in 0.3 m^3^ plastic cages at a constant temperature of 24 °C, with a 14:10 h L:D cycle. Each cage contained an oviposition cup with egg paper and multiple cotton stoppered vials containing 20% sucrose solution. Colonies were blood fed weekly from mice or guinea pigs anesthetized with a 9:1 ketamine:xylazine mixture (Illinois State University IACUC protocol #842,043). Egg papers were replaced weekly and stored at high humidity. See^46^ for further details on rearing.

### Overall experimental design

The experiment was run as a temporal block design with one complete replicate of population densities (experimental units) for a species constituting a temporal block. One day before hatching larvae, 1 L plastic containers received 1.0 g of dried white oak (*Quercus alba*) leaves and 0.10 g of dried, crushed crickets (*Gryllodes sigillatus*) as detritus sources that support the microbial assemblages that are food for larvae, and 900 mL of Nanopure® water. Containers were then incubated in an environmental chamber at 24 °C 14:10 h L:D cycle. These containers resemble typical aquatic systems colonized by these species in Florida and other parts of North America, where all three species occur in cemetery vases and tires, both in size and in detritus composition^56–58^. Each container was assigned a density treatment (10, 30, 50, 80, 110, 140, 170, 200, 250, or 300 first instar larvae) and a species treatment. Mosquito eggs from lab-reared colonies of *A. triseriatus, A. aegypti,* and *A. albopictus* were induced to hatch in Nanopure® water containing 0.25 g/L of bacteriological nutrient broth (Difco™), which provided newly hatched larvae with a food source for approximately 24 h until transfer to experimental containers. Twenty-four hours after initiating hatching, larvae were rinsed with Nanopure® water, counted into the 10 density treatments, and added to the containers. Containers were then returned to the environmental chamber and held under the same conditions.

Containers were checked daily for pupae by pouring into a shallow 30 × 15 cm pan. Pupae were placed into 0.92 mL glass vials with cotton stoppers and allowed to emerge as adults. Pans were then rinsed back into the 1 L containers and any evaporated or lost water replaced with Nanopure® water. After adults eclosed, residual water was removed from the vial, sexes determined, and date of eclosion recorded before being placed in a drying oven with temperature set to 50 °C for more than 24 h before further processing. Wing length of females was measured by placing removed wings on a microscope slide with coverslip to flatten for imaging. Images of each wing were taken using Epiphan Capture Tool (Epiphan Systems Inc. version 3.30.2.10) with a Wild M32 microscope and Sony DXC-970MD series microscope camera. Wing lengths were measured from the images using *ImageJ* software (National Institute of Health version 1.50i). Wing lengths were used to predict female fecundity using regressions (see below).

### Index of performance

The composite index of performance *r*’^36^ was used to estimate *per capita* rate of change for each replicate cohort for each of the species.

1 $$r^{\prime} = \frac{{\ln \, \left[ { \, \left( {1/N_{0} } \right){\sum_x} A_{x} f\left( {w_{x} } \right) \, } \right]}}{{D + \, \left[ {{{ \, \sum_x}} x A_{x} f\left( {w_{x} } \right)/{ \sum_x} A_{x} f\left( {w_{x} } \right) \, } \right]}}$$

where *N*_0_ is the initial number of females in the cohort (assumed to be ½ the initial number of larvae, as has been done in all previous applications of this index:^5,24,36,45–53,55^), *x* is number of days since hatching, *A*_*x*_ is the number of females from the cohort eclosing as adults on day *x, w*_*x*_ is mean wing length of females from the cohort eclosing on day *x*, *f*(*w*_*x*_) is predicted number of female eggs produced by females with mean wing length *w*_*x*_ based on regressions (Supplemental Table S2), and *D* is the estimated number of days from eclosion to oviposition (Supplemental Table S2). This index combines these measurements in a manner that is similar to life table estimates of *R*_0_ (net reproductive rate) and *T*_*c*_ (cohort generation time), allowing for estimation of population growth, estimated as *r* = ln(*R*_0_)/*T*_*c*_^36^, for a cohort of larvae when following reproduction of multiple replicated cohorts is infeasible. One problem with *r’* is that when no females survive to adulthood, either because of negative effects of high larval density (e.g., 300 larvae) or because of stochastic variation in number of females in small cohorts (e.g., 10 larvae), *r’* is not estimable. These cohorts cannot be ignored, particularly when larval density is high, as lack of survivors is then likely a result of high density. This problem can be solved by pooling replicate cohorts of the same density and species with and without surviving females to yield a combined cohort with larger *N*_*0*_, but having been reared at the nominal larval densities and *per capita* resources^24^. Thus, when no females survive in our data set this is the approach that we take. When data from two or more replicate cohorts at the same initial density were combined, we designate the combined data as coming from a new replicate block, identified with the numbers from the original blocks that were combined (e.g., combining cohorts from replicate blocks 1 and 3 yields a new replicate block 13).

### Statistical analysis

Relationships between *r*’ and initial density (*N*) were fit using the SAS statistical software (version 9.3) of the SAS system for windows (copyright © 2011 SAS Institute Inc). The fits included a θ logistic relationship, a Gompertz relationship with two shape parameters^59^, and linear, quadratic, and cubic relationships (Supplemental Table S3), all implemented using a generalised nonlinear mixed model (PROC NLMIXED), with a normal distribution of error and an identity link function, with replicate block as a random effect. The replicate block effect was modeled as affecting each of the parameters of the models (Supplemental Table S3). Corrected Akaike Information Criterion (AICc) values were calculated and used to determine which relationship provided the best description of the data^60^.

Relationships of survival to adulthood to initial density were analysed by fitting a generalised nonlinear mixed model (PROC NLMIXED), with a binomial distribution of error and logit link function, with replicate block as a random effect, of surviving adults (*S*) vs. initial density (*N*)^61,62^:

2 $$S = aN/\left[ {1 + \left( {N/K} \right)^{d} } \right]$$

The parameters *a*, *K*, and* d* in this phenomenological model determine the shape of the relationship of *S* to *N.* When the parameter *d* > 1.0, *S* peaks at a low *N* and declines at high *N* (specifically *N* ≥ *K*). When *d* = 1*.*0, *S* approaches an asymptote equal to *aK* as *N* increases. When *d* < 1.0, *S* is a monotonic increasing function of *N*. When *d* = 0, *S* increases linearly with *N.* The parameter *a* is interpreted as the proportion surviving as *N* approaches 0 (i.e., as *N* → 0, *S/N* → *a*). When *N* = *K*, *S* = *aN*/2. Different models included and tested for random variation among replicate blocks in each of the parameters *a*, *K,* and *d* in Eq. (2). Because the number of replicate containers at each *N* was small, only models postulating a random effect of replicate block on one parameter (*a*, *K*, or *d*) at a time, or no parameters were tested. Attempts to fit models with random variation in multiple parameters at the same time resulted in either nonconvergence of the iterative solution, or inestimable parameters. Best models for each species were determined by AICc.

Adult female development time (= median days to eclosion from a replicate container) and female size (= mean wing length from a replicate container) were analysed by generalised linear model ANOVA (SAS PROC GLIMMIX) using initial density *N* as a class variable with block as a random effect. For female mean wing length and female median development time the models used normal and negative binomial distributions of error, respectively, and identity and log link functions, respectively. Significant ANOVA results were followed by Tukey pairwise multiple comparisons among least-squares means.

### Ethical use of vertebrate animals

Use of live vertebrate animals, female mice [ICR (CD1) strain)] and male guinea pigs [Hartley strain], for blood feeding mosquito populations is approved by Illinois State University’s Institutional Animal Care and Use Committee (IACUC protocol #842043). Individuals working with live vertebrate animals were trained on the procedures outlined in this protocol and underwent additional webinar training. All experiments were performed in accordance with the guidelines and regulations for IACUC protocol #842043, and the relevant methods relating to the use of these live animals are in accordance with ARRIVE guidelines.

## Results

### *r’* vs. initial density

**Figure 1:**
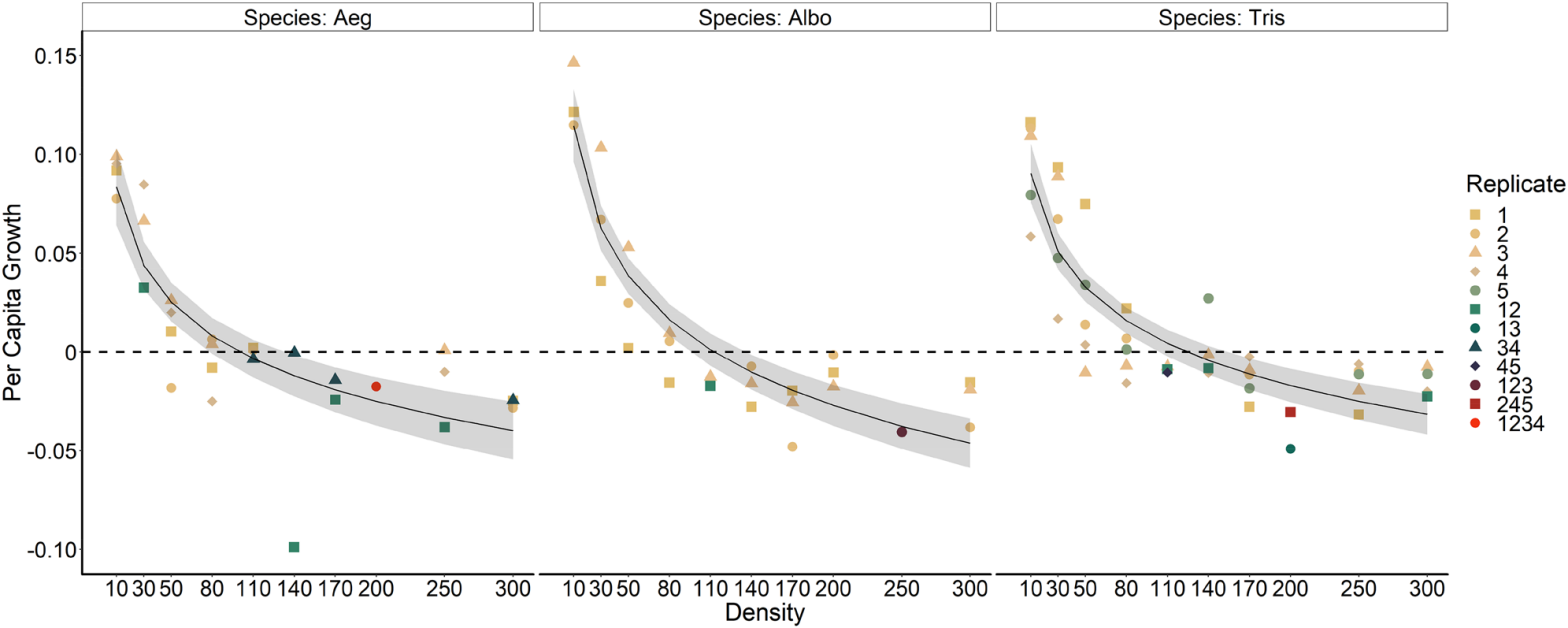
Relationship between estimated *per capita* rate of change *r’* and initial density *N* for *Aedes aegypti, Aedes albopictus,* and *Aedes triseriatus*, as described by the best models (Table 1), which were Gompertz models without random variation due to blocks. The solid black line is the predictions from the model averages, and the grey area represents 95% maximum likelihood confidence interval on the prediction (SAS PROC NLMIXED). Best fit parameters (± SE) for *A. aegypti, A. albopictus,* and *A. triseriatus* were: *r*_*0*_ = 0.167 ± 0.017, 0.223 ± 0.019, and 0.173 ± 0.014, and *b* = *− *0.0363 ± 0.0038, − 0.0472 ± 0.0041, and − 0.0358 ± 0.0032, respectively, respectively. The shape and color of data points corresponds to the different replicate blocks for each species; cases of combined cohorts from different blocks are indicated with multiple numbers (e.g., replicate blocks 13, 123, etc).

Gompertz models with no random effect for block for either parameter (*r*_*0*_ or *b*) proved to be the most plausible model of the relationship of *r’* to initial density *N* for each of the three *Aedes* species, based on AICc (Table 1). The best 3 functions in each case were always alternative versions of the Gompertz or Quadratic function (Table 1) and all linear functions had ΔAICc > 20 and model weights < 10^–4^, usually much less (Table S3). The results clearly and strongly refute the hypothesis of a linear relationship and are consistent with the hypothesis of a nonlinear relationship that is concave upward (Fig. 1). Best fit parameters of the Gompertz equation for each of the three species (Fig. 1) were similar and did not differ significantly in combined analysis.

**Table 1:** AICc values comparing the top three models of *r’* vs. initial density *N* for *Aedes aegypti, Aedes albopictus,* and *Aedes triseriatus.*

Model: parameter affected by random replicate block	Total parameters	AICc	ΔAICc	e^(−0.5^*^ΔAICc)^	Model Weight	Evidence ratio
***Aedes aegypti***	*n* = 29					
**Gompertz, no random var**	**3**	**− 127.5**	**0**	**1**	**0.707**	**1**
Gompertz, random *b*	4	− 125.0	2.5	0.287	0.202	3.49
Quadratic, no random var	4	− 123.4	4.1	0.129	0.091	7.77
***Aedes albopictus***	*n* = 27					
**Gompertz, no random var**	**3**	**− 127.5**	**0**	**1**	**0.989**	**1**
Quadratic, no random var	4	− 117.6	9.9	0.007	0.007	141.17
Gompertz, random *b*	4	− 116.3	11.2	0.004	0.004	270.43
***Aedes triseriatus***	*n* = 43					
**Gompertz, no random var**	**3**	**− 202.9**	**0**	**1**	**0.725**	**1**
Gompertz, random *r*_0_	4	− 200.5	2.4	0.301	0.218	3.32
Quadratic, no random var	4	− 197.6	5.3	0.071	0.051	14.15

Models that failed to converge excluded, and those beyond the top three are included in the supplemental material (Table S2). For each species the best model is highlighted in bold face type. Functions given in Supplemental Table S4. Within the table ΔAICc is the difference between AICc on the current line and AICc for the top line (i.e., the most plausible model).

### Surviving adults vs. initial density

**Figure 2:**
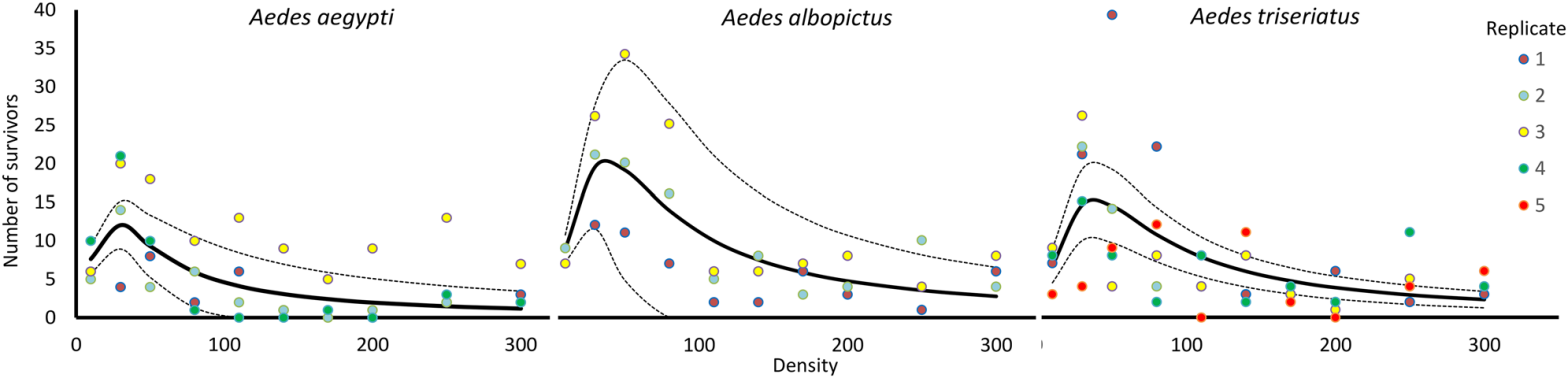
Survivorship to adulthood *S* versus initial density *N* for *Aedes aegypti. Aedes albopictus,* and *Aedes triseriatus*. The solid black line is the prediction for the regression of number of surviving adults vs. density (Eq. 2) removing effects of random variation among blocks, and the dotted lines indicate 95% maximum likelihood confidence interval on that prediction (SAS PROC NLMIXED), also removing the effects of random variation among blocks. Because *S* cannot be less than 0 the graph is truncated at 0 survivors; some lower confidence limit estimates reached values of ~ -1. Best fit parameters for *A. aegypti, A. albopictus,* and *A. triseriatus* are given in Table 3. The color of data points corresponds to the different replicate blocks for each species.

The nonlinear relationship from Eq. (2) generally fit the data on surviving adults vs. initial density (Fig. 2), but the three species differed in how the random effect of replicate block affected the relationship, with each species’ best model having a different parameter impacted by that random variation (Table 2). The best nonlinear description for each species yielded *d* significantly greater than 1.0 (Table 3), indicating a peak in adult production at low density and lesser adult production at greater densities (Fig. 2). Estimates of *d* for the three species were similar, falling between 2.30 and 2.37 (Table 3) indicating a fairly sharp decline in adult production as density increased (Fig. 2). The species differed more in *a* and *K*, with *a* lowest for *A. triseriatus* and greatest for *A. albopictus*, and *K* lowest for *A. aegypti* and similar for *A. albopictus* and *A. triseriatus* (Table 3).

**Table 2:** AICc values comparing models of surviving adults *S* vs. initial density *N* for *Aedes aegypti, Aedes albopictus,* and *Aedes triseriatus.*

Model: parameter affected by random replicate block	Total parameters	AICc	ΔAICc	e^(−.5^*^ΔAICc)^	Model weight	Evidence ratio
***Aedes aegypti***						
***d* random**	**5**	**225.0**	**0**	**1**	**0.802**	**1**
*K* random	5	227.8	2.8	0.247	0.198	4.06
*a* random	5	260.1	35.1	2.39 × 10^–8^	1.91 × 10^–8^	4.19 × 10^7^
no random effects	4	292.7	67.7	1.99 × 10^–15^	1.60 × 10^–15^	5.02 × 10^14^
***Aedes albopictus***						
***K* random**	**5**	**184.8**	**0**	**1**	**0.996**	**1**
*a* random	5	195.7	10.9	0.004	0.004	232.76
*d* random	5	211.7	26.9	1.44 × 10^–6^	1.44 × 10^–6^	6.94 × 10^5^
no random effects	4	213.1	28.3	7.16 × 10^–7^	7.13 × 10^–7^	1.40 × 10^4^
***Aedes triseriatus***						
***a* random**	**5**	**386.6**	**0**	**1**	**0.997**	**1**
*K* random	5	398.2	11.6	0.003	0.003	330.30
no random effects	4	408.8	22.2	1.51 × 10^–5^	1.51 × 10^–5^	66,171.16
*d* random	5	411.1	24.5	4.79 × 10^–6^	4.77 × 10^–6^	2.09 × 10^5^

Models that failed to converge excluded. For each species the best model is highlighted in bold face type. Within the table ΔAICc is the difference between AICc on the current line and AICc for the top line (i.e., the most plausible model).

**Table 3:** Best fit parameters for the relationships of surviving adults *S* vs. initial density *N* (Eq. 2) for *Aedes aegypti, Aedes albopictus, and Aedes triseriatus* that are shown in Fig. 2.

Species (*n* replicate blocks)	Parameter	Estimate ± SE	95% confidence interval
*Aedes aegypti* (4)	*a*	0.83 ± 0.07	0.59–1.06
	*K*	29.2 ± 3.0	19.5–38.8
	d*	2.30 ± 0.29	1.37–3.23
*Aedes albopictus* (3)	*a*	0.92 ± 0.04	0.73–1.12
	K*	43.0 ± 5.8	18.2–67.8
	*d*	2.37 ± 0.13	1.82–2.91
*Aedes triseriatus* (5)	a*	0.69 ± 0.09	0.46–0.93
	*K*	43.1 ± 3.3	31.1–52.2
	*d*	2.32 ± 0.12	1.99–2.64

See Table 2 for model selection via AICc. For each species the parameter that varied among replicate blocks is indicated with an *.

### Development time and wing length vs. initial density

**Figure 3:**
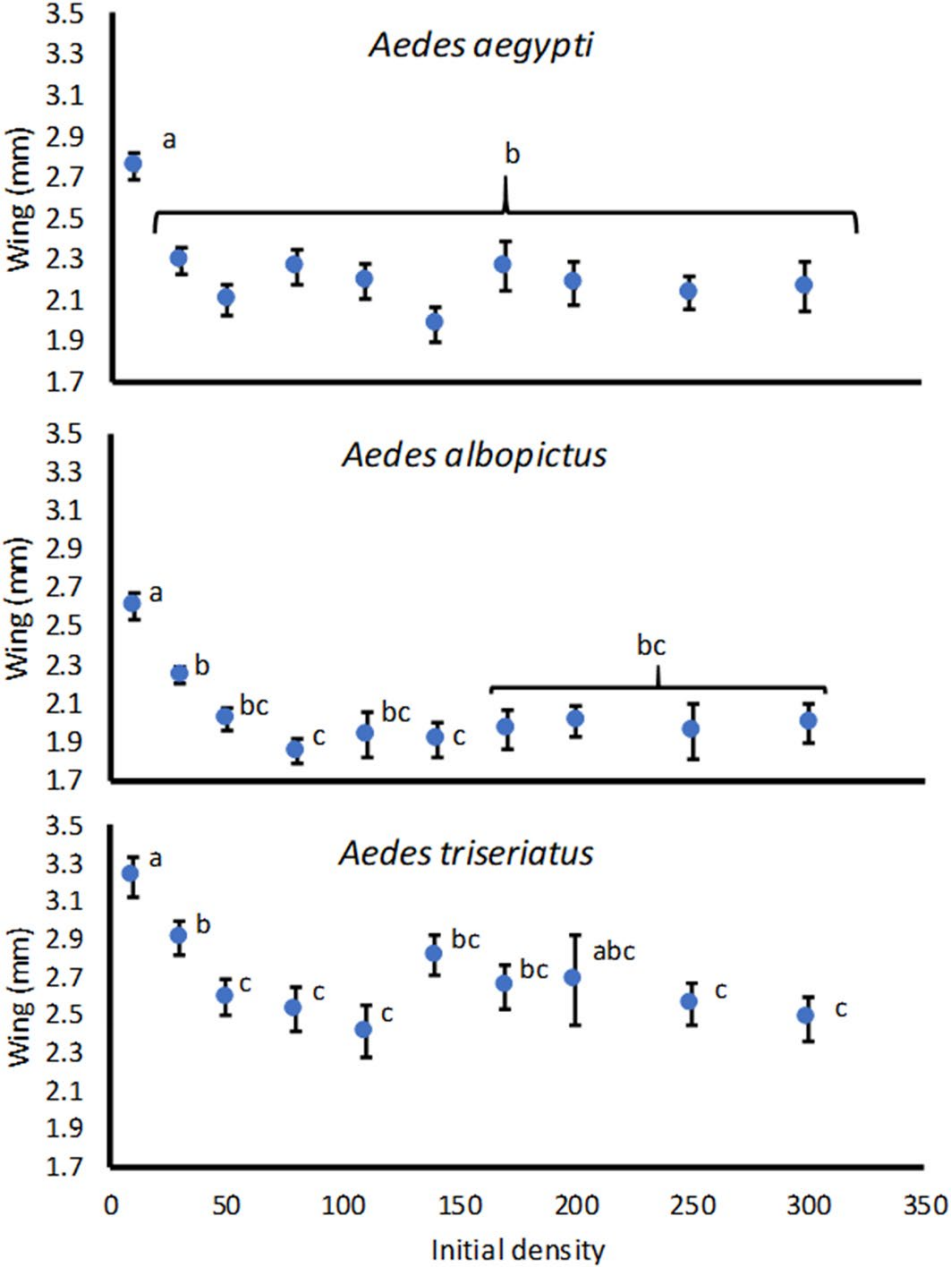
Least squares mean ± SE of cohort mean female wing length for *Aedes aegypti, Aedes albopictus,* and *Aedes aegypti,* averaged over replicate runs for each species. Means for a species associated with the same letters are not significantly different by Tukey’s multiple comparisons.

Female size was significantly affected by initial density for *A. aegypti* (F_9,13_ = 7.43, P = 0.0007), *A. albopictus* (F_9,14_ = 21.98, P < 0.0001), and *A. triseriatus* (F_9,25_ = 5.72, P = 0.0003). All three species showed a pattern of greatest mean female wing length at the lowest density of 10 larvae and statistically indistinguishable mean wing lengths at all densities ≥ 50 larvae, with only the mean at density of 200 *A. triseriatus* larvae not significantly different from the mean at density of 10 *A. triseriatus* larvae (Fig. 3).

**Figure 4:**
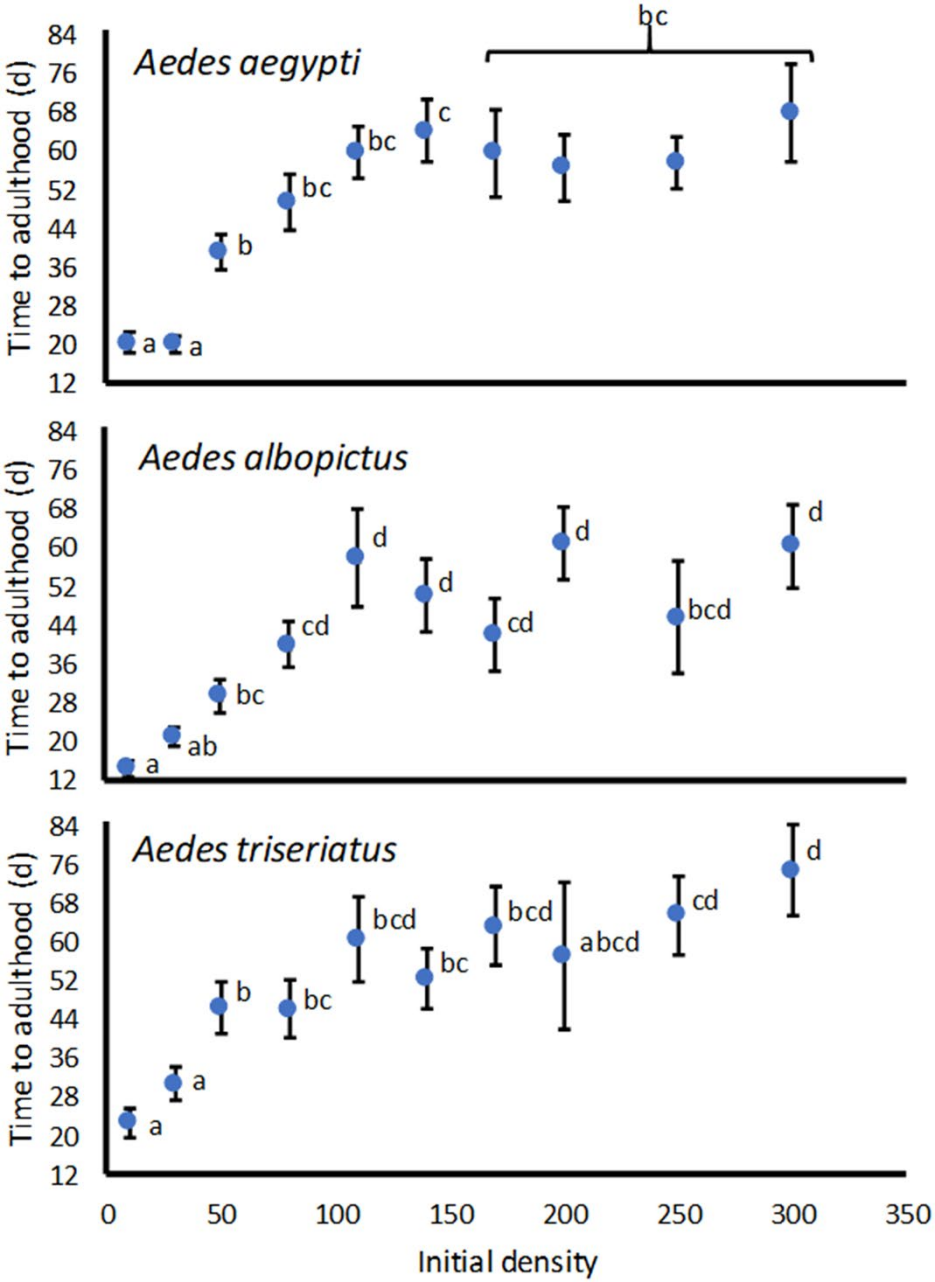
Least squares mean ± SE of cohort median female time to adulthood for *Aedes aegypti, Aedes albopictus,* and *Aedes aegypti,* averaged over replicate runs for each species. Least squares means for a species associated with the same letters are not significantly different by Tukey’s multiple comparisons

Female development time was significantly affected by initial density for *A. aegypti* (F_9,14_ = 7.40, P = 0.0005), *A. albopictus* (F_9,14_ = 7.48, P = 0.0005), and *A. triseriatus* (F_9,26_ = 9.55, P < 0.0001). All three species showed a pattern of lowest and statistically indistinguishable mean female days to adulthood at the two lowest densities, steadily increasing mean days to adulthood at densities of 50 and 80 larvae, and most means for days to adulthood significantly greater at densities ≥ 100 larvae than at densities ≤ 30 larvae (Fig. 4). Most means of days to adulthood were statistically indistinguishable among densities ≥ 110 larvae (Fig. 4).

## Discussion

We found that the relationship between density and the estimated *per capita* rate of increase is clearly non-linear, as the most plausible models for our data were in all three cases the Gompertz function, with no random variation among replicate blocks. In the cases of *A. aegypti* and *A. triseriatus* the second-most plausible models, by AICc, were in both cases Gompertz functions with random variation in the parameter *b*, but these models only attainted model weights of ~ 0.21 indicating they were rather unlikely to be the correct model for these data. All other models yielded very small model weights (< 0.10; Table 1). All linear models tested had model weights < 10^–6^_._ Thus, these results are inconsistent with the common assumption of logistic growth, that rate of increase declines linearly with density. Because we used information criteria to compare different models for these data, we do not claim that the Gompertz model is the true model that generated the data, only that it is the most plausible model among those we tested^60^, and is sufficient to show the nonlinearity in the relationship of *r’* to initial density *N.* Our analysis is one of just a few experimental tests of the assumption of linearity of rate of increase vs. density in animal model systems^11,37,41–43,53^.

Our estimates of *per capita* growth rate are derived from components of: survivorship to adulthood; female development time to adulthood (indicating age at reproduction); and adult female size (indicating fecundity), all combined in an approach similar to standard life table calculations^36^. All these components were strongly nonlinearly related to initial population density. The relationships of *per capita* growth to density were all concave upward, indicating a rapid decline in growth rate across a range of low densities, and a slowing of the decline as densities become greater (Fig. 1). This pattern of density dependent growth thus seems to be associated with relatively little change in number of adults produced, female development time, and female size over a range of high densities, contrasting with the rapid change in all components as densities rise from the lowest levels in the experiment (Figs. 2, 3, 4).

Analysis of individual based models of a single species competing for resources gave insight into how life history and biological traits of organisms produce nonlinearity in *per capita* growth^4^. That research showed that both scramble and contest competition for resources could result in nonlinear relationships of *per capita* growth to density, and that whether that relationship was concave upward, or concave downward depended on maximum individual reproductive output, and the range of resource consumption over which reaching reproductive maturity is possible (i.e., plasticity in response to resource consumption). Greater maximum fecundity, and greater plasticity in response to resource consumption (specifically ability to reproduce at low resource intake) produced a relationship of *per capita* growth vs. population density that is concave upward, whereas lower maximum fecundity and lesser plasticity in response to resource consumption (specifically a high minimum resource intake requirement) produced a relationship of *per capita* growth vs. population density that is concave downward^4^. For the *Aedes* species we investigated, individual maximum fecundity can be moderately high (i.e., 50–100 eggs per reproductive cycle; See Fig. 3 for mean sizes, and Supplemental Table S2, for size fecundity relationships), but these *Aedes* are well known for their high plasticity in development time to and size at adulthood in response to food availability and competition^63–66^ and ability to survive and to complete development with very low food availability^67^. These same traits likely contribute to the concave relationships we observed, and in particular the shallow decline in *per capita* growth over the high experimental densities.

The non-linearity of the relationship of adults produced vs. density indicates that overcompensatory morality is likely^68,69^ and has been detected with experimental mortality in the laboratory for all three species^5,46^. Overcompensation occurs when extrinsic mortality imposed on a population decreases density and negative effects of density on survival, resulting in increased adult production with mortality. The estimated values of the parameter *d* in Eq. (2) are all significantly > 1.0, which is indicative of overcompensation in response to mortality^61,62^. This situation, if it occurs for these species in nature, could be important for mosquito control in the interests of public health^26^. Historically, pest management has targeted populations of pests with limited attention to how mortality interacts with density dependence to affect population dynamics^70^. The results from our experiments show that reductions of larval populations could lead to greater production of adults, in addition to increased *per capita* growth rates. Because of the variability in our data, we cannot make a confident estimate of the initial density of any of the species that would yield maximum adult production, but our data appear consistent in placing that peak among the lower initial densities that we used. Variation among replicate runs in numbers of surviving adults was considerable (Fig. 2) and was identified as an important element for nonlinear models of density dependent survival (Tables 2 and 3). That variation seems greatest for *A. triseriatus*, resulting in the average model for *A. triseriatus* (Fig. 2) describing the data less well than those for the other species. Regardless of the source of this variation among replicates, incorporating it into our models seems to have been important for detecting the fixed effect of density on number of survivors.

Here we have demonstrated that the relationship between density and estimated *per capita* growth for these mosquitoes is non-linear. As models postulating a linear relationship have often been used with these mosquitoes to quantify expected effects of intra- and inter-specific competition^24,36,45,47–53^ our results raise the question: what conclusions from using linear models may be misleading? If data are from experiments at relatively low densities, relative to resources (i.e., densities ≤ 80 larvae in our experiments; Fig. 1), it is likely that a linear relationship would be a fairly accurate approximation of the effects of intraspecific competition (i.e., estimating carrying capacity) or interspecific competition (i.e., estimating interspecific competition coefficients). The concave upward shape of the curves in Fig. 1 indicate that carrying capacity will be underestimated by such a linear approximation. In contrast, if data are from relatively high densities relative to resources (i.e., densities ≥ 140 larvae in our experiment; Fig. 1), the shallow slope in this range is likely to yield underestimates of the maximum *per capita* rate of increase for a population at low density. This suggests that populations of these *Aedes* species have a great potential for rapid increases from rarity caused by reductions via mosquito control or associated with being a newly introduced species. *Aedes* in general, and *A. albopictus* and *A. aegypti* in particular, have been among the most successful invasive species worldwide^71,72^, and this suggestion of very high potential population growth when rare seems likely to be a contributing factor to this invasion success. When experimental data are from a wide range of densities relative to resources, using linear models is most likely to yield inaccurate estimates of population growth parameters. Further, the concave upward shape of this relationship suggests that populations may have approximately 0 growth over a relatively wide range of high densities.

## Supplementary Information

Supplementary Tables.

## Data Availability

The population level data for each container generated from this experiment will be available at Figshare.
