# Supplementary material for: Non-linear relationships between density and demographic traits in three Aedes species
Source: Sci Rep. 2022 May 16;12:8075. doi: 10.1038/s41598-022-11909-y (PMC9110365; doi:10.1038/s41598-022-11909-y)
Supplement: Supplementary file 1 — Supplementary Tables. [file 41598_2022_11909_MOESM1_ESM.docx]

**Supplemental Table S1.**  Origins of colonies of *Aedes* species used in the experiment. All sites USA.

| Species | Collection site | Block | Hatch date for experiment |
| --- | --- | --- | --- |
| *Aedes aegypti* | New Orleans LA | 1 | 16 July 2016 |
| *Aedes aegypti* | New Orleans LA | 2 | 4 August 2016 |
| *Aedes aegypti* | Vero Beach FL | 3 | 20 October 2016 |
| *Aedes aegypti* | Vero Beach FL | 4 | 13 June 2017 |
| *Aedes albopictus* | Tyson Research Center, Eureka MO | 1 | 4 August 2016 |
| *Aedes albopictus* | Tyson Research Center, Eureka MO | 2 | 1 January 2017 |
| *Aedes albopictus* | Orlando FL | 3 | 13 June 2017 |
| *Aedes triseriatus* | Tyson Research Center, Eureka MO | 1 | 30 September 2015 |
| *Aedes triseriatus* | Tyson Research Center, Eureka MO | 2 | 16 November 2015 |
| *Aedes triseriatus* | Tyson Research Center, Eureka MO | 3 | 24 January 2016 |
| *Aedes triseriatus* | Tyson Research Center, Eureka MO | 4 | 13 July 2016 |
| *Aedes triseriatus* | Tyson Research Center, Eureka MO | 5 | 4 August 2016 |

**Supplemental table S2.** Regression relationships of eggs laid (*E*) vs. wing length (*w* in mm) for each *Aedes* species in the experiment, used to obtain *f* (*w_x_*) to calculate *r’.* *D* is the estimate of the number of days from eclosion to oviposition. All relationships are for total eggs and were multiplied by 0.5 to predict female eggs.

| Species | Regression for *f* (*w_x_*) | *n* obs. | *r*^2^ | Regression Source | *D* |
| --- | --- | --- | --- | --- | --- |
| *Aedes aegypti* | *E =* 40.694(*w*) - 48.739 | 536 | 0.12 | Chandrasegaran & Juliano 2010 | 12 |
| *Aedes albopictus* | *E =* 78.02(*w*) -121.24 | 115 | 0.81 | Lounibos et al. 2002 | 14 |
| *Aedes triseriatus* | *E* = 1.8544(*w*^3.0024^) | 36 | 0.55 | Aspbury & Juliano 1999 | 12 |

Chandrasegaran, K, SA Juliano. 2019. How do trait-mediated non-lethal effects of predation affect population-level performance of mosquitoes? Frontiers in Ecology & Evolution, 7:25.

Lounibos, LP, RL Suárez, Z Menéndez, N Nishimura, RL Escher, SM O’Connell, JR Rey. 2002. Does temperature affect the outcome of larval competition between *Aedes aegypti* and *Aedes albopictus*? Journal of Vector Ecology 27: 86–95.

Aspbury, AS, Juliano, SA. 1998. Negative effects of drying and prior exploitation on the detritus resource in an ephemeral aquatic habitat. Oecologia 115:137-148

**Supplemental table S3.** AICc values comparing models of *r’* vs. initial density *N* for *Aedes aegypti, Aedes albopictus,* and *Aedes triseriatus.* Models that failed to converge excluded. For each species the best model is highlighted in **bold face type.** Functions given in Supplemental Table S4. Within the table Δ corresponds to difference.

| Model | Total Parameters | AICc | ΔAIC | e^(-.5*Δ)^ | Model Weight | Evidence ratio |
| --- | --- | --- | --- | --- | --- | --- |
| *Aedes aegypti* | *n*=29 |  |  |  |  |  |
| **Gompertz, no random var.** | **3** | **-127.5** | **0** | **1** | **0.707** | **1** |
| Gompertz, random *b* | 4 | -125.0 | 2.5 | 0.287 | 0.202 | 3.49 |
| Quadratic, no random var. | 4 | -123.4 | 4.1 | 0.129 | 0.091 | 7.77 |
| Linear, no random var. | 3 | -107.2 | 20.3 | 3.91x10^-5^ | 2.76x10^-5^ | 2.56x10^4^ |
| Gompertz, random *r_0_* | 4 | -97.3 | 30.2 | 2.77x10^-7^ | 1.96x10^-7^ | 3.61x10^6^ |
| Linear, random *r_0_* | 4 | -95.6 | 31.9 | 1.18x10^-7^ | 8.36x10^-8^ | 8.45x10^6^ |
| Linear, random *b* | 4 | -87.8 | 39.7 | 2.39x10^-9^ | 1.69x10^-9^ | 4.18x10^8^ |
| Cubic, random *d* | 6 | -81.7 | 45.8 | 1.13x10^-10^ | 8.01x10^-11^ | 8.82x10^9^ |
| Quadratic, random *c* | 5 | -62.7 | 64.8 | 8.49x10^-15^ | 6.00x10^-15^ | 1.18x10^14^ |
| Cubic, no random var. | 5 | -56.3 | 71.2 | 3.46x10^-16^ | 2.44x10^-16^ | 2.89x10^15^ |
| Cubic, random *c* | 6 | -52.6 | 74.9 | 5.44x10^-17^ | 3.84x10^-17^ | 1.84x10^16^ |
| Cubic, random *r_0_* | 6 | 9.7 | 137.2 | 1.61x10^-30^ | 5.35x10^-30^ | 1.32x10^29^ |
| *Aedes albopictus* | *n=*27 |  |  |  |  |  |
| **Gompertz, no random var.** | **3** | **-127.5** | **0** | **1** | **0.989** | **1** |
| Quadratic, no random var. | 4 | -117.6 | 9.9 | 0.007 | 0.007 | 141.17 |
| Gompertz, random *b* | 4 | -116.3 | 11.2 | 0.004 | 0.004 | 270.43 |
| Gompertz, random *r_0_* | 4 | -106.5 | 21.0 | 2.75x10^-5^ | 2.72x10^-5^ | 3.63x10^4^ |
| Linear, no random var. | 3 | -96.9 | 30.6 | 2.27x10^-7^ | 2.24x10^-7^ | 441x10^6^ |
| Linear, random *r_0_* | 4 | -90.5 | 37.0 | 9.24x10^-9^ | 9.1x10^-9^ | 1.08x10^8^ |
| Linear, random *b* | 4 | -72.6 | 54.9 | 1.20x10^-12^ | 1.19x10^-12^ | 8.34x10^11^ |
| Cubic, random *d* | 6 | -66.4 | 61.1 | 5.40x10^-14^ | 5.34x10^‑14^ | 1.85x10^13^ |
| Quadratic, random *c* | 5 | -55.5 | 72.0 | 2.32x10^-16^ | 2.29x10^-16^ | 4.31x10^15^ |
| Cubic, no random var. | 5 | -45.5 | 82.0 | 1.56x10^-18^ | 1.55x10^-18^ | 6.40x10^17^ |
| Cubic, random *c* | 7 | -37.3 | 90.2 | 2.59x10^-20^ | 2.56x10^-20^ | 3.86x10^19^ |
| Cubic, random *r_0_* | 7 | 13.2 | 140.7 | 2.80x10^-31^ | 2.77x10^-31^ | 3.57x10^30^ |
| *Aedes triseriatus* | *n*=43 |  |  |  |  |  |
| **Gompertz, no random var.** | **3** | **-202.9** | **0** | **1** | **0.725** | **1** |
| Gompertz, random *r*_0_ | 4 | -200.5 | 2.4 | 0.301 | 0.218 | 3.32 |
| Quadratic, no random var. | 4 | -197.6 | 5.3 | 0.071 | 0.051 | 14.15 |
| Gompertz, random *b* | 4 | -193.2 | 9.7 | 0.008 | 0.006 | 127.74 |
| Linear, no random var. | 3 | -173.0 | 29.9 | 3.22x10^-7^ | 2.33x10^-7^ | 3.11x10^6^ |
| Linear, random *r*_0_ | 4 | -172.9 | 30.0 | 3.06x10^-7^ | 2.212x10^-7^ | 3.27x10^6^ |
| Linear, random *b* | 4 | -136.1 | 66.8 | 3.12x10^-15^ | 2.26x10^-15^ | 3.20x10^14^ |
| Cubic, random *d* | 6 | -131.1 | 71.8 | 2.56x10^-16^ | 1.89x10^-16^ | 3.90x10^15^ |
| Quadratic, random *c* | 5 | -100.3 | 102.6 | 5.26x10^-23^ | 3.81x10^-23^ | 1.90x10^22^ |
| Cubic, no random var. | 5 | -96.0 | 106.9 | 6.12x10^-24^ | 4.44x10^-24^ | 1.63x10^23^ |
| Cubic, random *c* | 6 | -83.8 | 119.1 | 1.37x10^-26^ | 9.95x10^-27^ | 7.28x10^25^ |

**Supplemental table S4.** Basic regression relationships used for fitting models to the relationship of *r’* (estimated *dN/Ndt* or *per capita* rate of change) vs. *N* (initial number of larvae).

| Function | Equation |
| --- | --- |
| Gompertz | *r’* = *r*_0_ + *b*[ ln(*N*)] |
| Cubic | *r’* = *r*_0_ [1 + *bN* + *cN*^2^ + *dN*^3^ ] |
| Quadratic | *r’* = *r*_0_ [1 + *bN* + *cN*^2^ ] |
| Logistic (Linear) | *r’* = *r*_0_ [*K* – *N*]/*K* = *r*_0_ [1 – (*N*/*K*)] |
| θ Logistic | *r’* = *r*_0_ [1 – (*N*/*K*)^θ^] |
